# Role of Gut and Urinary Microbiome in Children with Urinary Tract Infections: A Systematic Review

**DOI:** 10.3390/diagnostics15010093

**Published:** 2025-01-03

**Authors:** Anjali Srivastava, Omprakash Shete, Annu Gulia, Sumit Aggarwal, Tarini Shankar Ghosh, Vineet Ahuja, Sachit Anand

**Affiliations:** 1 Department of Pediatric Surgery, All India Institute of Medical Sciences, New Delhi 110029, India; srivastavaanjali2324@gmail.com (A.S.); annugulia664@gmail.com (A.G.); 2 Department of Computational Biology, Indraprastha Institute of Information Technology Delhi (IIIT-Delhi), Okhla Phase III, New Delhi 110020, India; omprakashs@iiitd.ac.in (O.S.); tarini.ghosh@iiitd.ac.in (T.S.G.); 3 Descriptive Research, Indian Council of Medical Research, New Delhi 110029, India; drsumit.ecd@gmail.com; 4 Department of Gastroenterology and Human Nutrition, All India Institute of Medical Sciences, New Delhi 110029, India; vins_ahuja@hotmail.com

**Keywords:** urinary tract infections, microbiome, microbiota, gut microbiome, urinary microbiome, urobiome, dysbiosis, children

## Abstract

**Background**: The complex interaction between the gut and urinary microbiota underscores the importance of understanding microbial dysbiosis in pediatric urinary tract infection (UTI). However, the literature on the gut–urinary axis in pediatric UTIs is limited. This systematic review aims to summarize the current literature on the roles of gut and urinary dysbiosis in pediatric UTIs. **Methods**: This systematic review was conducted in accordance with the Preferred Reporting Items for Systematic Reviews and Meta-Analyses guidelines. A comprehensive literature search was performed across four databases, including PubMed, Web of Science, Scopus, and EMBASE. All studies published between January 2003 and December 2023 utilizing 16S rRNA sequencing to profile the gut or urinary microbiome in children with UTIs were included. Heat map visualization was used to compare microbial profiles between UTI and control cohorts. The methodological quality assessment was performed using the Newcastle–Ottawa scale (NOS). **Results**: Eight studies were included in this review. While five studies compared the microbiota signatures between patients and controls, three studies focused solely on the UTI cohort. Also, the gut and urinary microbiome profiles were investigated by four studies each. The consistent loss of microbiome alpha-diversity with an enrichment of specific putative pathobiont microbes was observed among the included studies. *Escherichia coli* consistently emerged as the predominant uropathogen in pediatric UTIs. In addition to this, *Escherichia fergusonii*, *Klebsiella pneumoniae*, and *Shigella flexneri* were isolated in the urine of children with UTIs, and enrichment of *Escherichia*, *Enterococcus*, *Enterobacter*, and *Bacillus* was demonstrated in the gut microbiota of UTI patients. On the contrary, certain genera, such as *Achromobacter*, *Alistipes*, *Ezakiella*, *Finegoldia*, *Haemophilus*, *Lactobacillus*, *Massilia*, *Prevotella*, *Bacteroides*, and *Ureaplasma*, were isolated from the controls, predominantly in the fecal samples. The methodological quality of the included studies was variable, with total scores (NOS) ranging from 5 to 8. **Conclusions**: The enrichment of specific pathobionts, such as *Escherichia coli*, in the fecal or urinary samples of the UTI cohort, along with the presence of core microbiome-associated genera in the non-UTI population, underscores the critical role of the gut–urinary axis in pediatric UTI pathogenesis. These findings highlight the potential for microbiome-based strategies in pediatric UTIs. Further studies with larger cohorts, standardized healthy controls, and longitudinal profiling are essential to validate these observations and translate them into clinical practice.

## 1. Introduction

Urinary tract infections (UTIs) are a significant health challenge, affecting approximately 8% of children at some point in their lives [1]. These infections, characterized by non-specific symptoms, pose a significant burden on healthcare systems and increase the risk of long-term complications if not detected and managed promptly. Traditionally, *E. coli* has been recognized as the primary uropathogen in pediatric UTIs [2]. However, recent advancements in microbiome research have identified a more intricate and specific microbial profile, suggesting that the etiology of UTIs may involve some other organisms and even a complex interplay between the gut and the urinary microbiomes [3].

The human microbiome plays a critical role in maintaining health and influencing disease processes, including UTIs [4]. Dysbiosis, or an imbalance in these microbial communities, has emerged as a key factor in susceptibility to various infections, including UTIs [5]. Sporadic studies have highlighted that the gut microbiome may serve as a potential reservoir for uropathogens, which may migrate to the urinary tract under certain conditions, leading to UTIs [6]. This migration and subsequent colonization of the urinary tract by microorganisms can explain the recurrent and chronic nature of UTIs observed in some pediatric patients [7].

The mechanisms by which gut and urinary microbiomes may interact to influence the risk of UTI are complex and multifaceted. First, dysbiosis in the gut leads to alterations in the gut microbial profiles and the functional capabilities of microbes, making the gut more permissive to uropathogen colonization. This facilitates the migration of pathogenic bacteria from the gastrointestinal tract to the urinary system, either through direct transit or hematogenous spread, particularly when the gut barrier function is impaired [8]. The latter may especially be observed during scenarios of impaired gut barrier function, leading to the translocation of pathogenic bacteria like *E. coli* from the gut to the blood. This process is often accompanied by metabolic shifts in the gut, affecting factors such as urinary pH and metabolite production, which further promote pathogen colonization in the urinary tract [9]. The second mechanism involves the gut acting as an agitator, where severe dysbiosis amplifies systemic inflammatory responses. This pro-inflammatory state, driven by microbial metabolites like short-chain fatty acids (SCFAs), can disrupt urinary tract defenses and promote pathogen persistence, increasing the likelihood of recurrent infections [8]. Finally, the gut may act as a passive reservoir for uropathogens, indirectly influencing UTI occurrence without directly affecting UTI risk [8].

The intricate relationship between the gut and the urinary microbiota underscores the importance of understanding microbial dysbiosis in pediatric UTIs. This is particularly crucial for children with congenital malformations of the urinary tract, who are at risk of developing UTIs due to the complex interplay of the host and the microbial factors. The aberrant host micro-environment, arising from the congenital defect in the urinary tract, along with a dysregulated host immune response and fecal–urinary dysbiosis, results in recurrent infections, prolonged hospital stays, elevated healthcare costs, and frequent recurrences [10].

Despite the critical role of the gut–urinary axis in pediatric UTIs, there is a dearth of literature on this aspect. The fecal and urinary microbiome in children with UTIs is scarcely investigated. As per our best understanding, this is the first systematic review that aims to summarize the current published literature on the roles of gut or urinary dysbiosis in pediatric UTIs, with a focus on identifying key microbial species; elucidating their pathogenic mechanisms; and evaluating how these dysbiotic profiles contribute to infection risk, severity, and recurrence. By virtue of this, we intend to enhance our understanding of microbiome–UTI interactions and explore their clinical implications.

## 2. Materials and Methods

### 2.1. Literature Search

This systematic review was conducted in adherence to the Preferred Reporting Items for Systematic Reviews and Meta-Analyses (PRISMA) guidelines [11]. The review was prospectively registered in PROSPERO to ensure methodological transparency [12]. On 15 May 2024, a comprehensive literature search was performed by two independent reviewers (AS and AG) across several databases, including PubMed, Web of Science, Scopus, and EMBASE. The search strategy was designed using a combination of keywords: “microbiome”, “microbiota”, “microbe”, “microorganism”, “dysbiosis” combined with “gut”, “fecal”, “urine”, “urinary”, and “urinary tract infections”, “UTI”, and “children”. The search syntax and detailed strategy are outlined in Appendix A. Duplicate entries were removed using Rayyan web reference management software [13], and the remaining records were screened for relevance.

### 2.2. Eligibility

The studies focusing on children aged ≤ 18 years of age with a diagnosis of UTI were eligible for inclusion. The inclusion criteria for this study encompassed research utilizing 16S rRNA sequencing to profile the gut or urinary microbiomes. All studies published between January 2003 and December 2023 were included. Studies were excluded if they involved patients with neurogenic bladder, indwelling urinary catheters, stents, or infections beyond UTIs or those using culture-based methods and/or reporting fewer than two isolated organisms or pathogens (e.g., *Corynebacterium* spp., *Lactobacillus* spp., and coagulase-negative *Staphylococci*). Preclinical research, reviews, non-English publications, case reports, conference abstracts, opinion pieces, and editorials were also excluded.

### 2.3. Microbial Data Extraction and Visualization

Microbial data extraction was performed manually by two investigators independently (AS and OS). The extracted data encompassed study details including author names, publication year, country of origin, study design, sample size, microbiome type (gut and urinary), and microbiome characteristics including alpha diversity, beta diversity, and specific microbial information. Discrepancies in data extraction were resolved through a consensus process involving a third reviewer (SA) to ensure accuracy and consistency.

A heat map was generated using the pheatmap package (version 1.0.12) in R by two authors (AS and OS) to depict the difference in microbial profiles among the included studies. Only studies that included microbial data for both UTI and control cohorts were used to generate the heat map.

### 2.4. Methodological Quality Assessment

The methodological quality of the included studies was evaluated using the Newcastle–Ottawa scale (NOS) [14]. The NOS assesses studies based on three domains: selection of study groups, comparability of groups, and ascertainment of outcomes or exposures [15]. Each study was scored according to the predefined criteria within these domains, providing a comprehensive assessment of the study quality.

## 3. Results

### 3.1. Characteristics of the Included Studies

**Figure 1:**
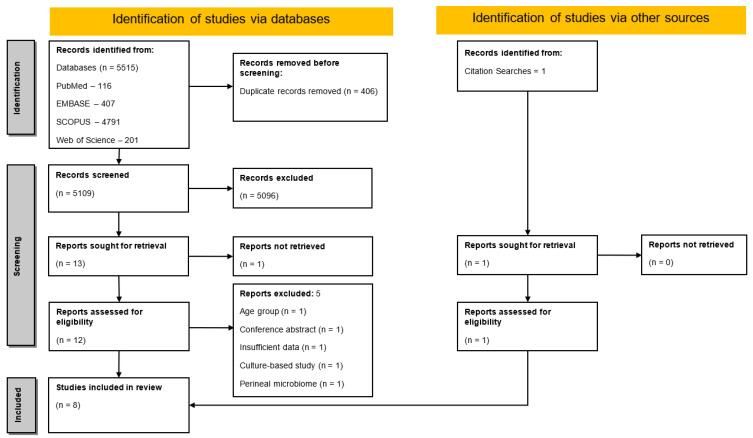
Flow diagram and study selection process as per the preferred reporting items for systematic reviews and meta-analyses (PRISMA) guidelines.

A total of 5515 records were initially identified, of which 406 were duplicates and removed (Figure 1). Upon subsequent screening of the remaining 5109 records, 5097 were not relevant to our study objectives and were thus excluded. Among the remaining twelve reports sought for retrieval, five were excluded due to the following reasons: one study encompassed individuals > 18 years of age [16]; one of them was a conference abstract, while one had insufficient data pertaining to the details of the investigations and microbiome abundance profiles [17,18]; one was a cross-sectional retrospective study focused on antibiotic resistance patterns of UTI pathogens identified using culture-based methods [19]; and one pilot study investigated the different perineal microbiome in children with UTI [20]. Along with these seven studies selected via the database search, one additional study was identified through alternative sources. Thus, a total of eight studies were included in the systematic review (Table 1).

Four of these studies [21,22,23,24] directly examined the microbiome in urine samples. While Choi et al. [21] and Marshall et al. [22] profiled uropathogens in urine from specifically pediatric UTI patients, providing insights into distinct pathogen prevalences in these patients, Kinneman et al. [23] adopted a case-control design, comparing the urine microbiomes from UTI patients with controls and identifying microbiome alterations between the two groups. Vitko et al. [24], on the other hand, investigated the variations in the microbial and metabolic profiles in cases of vesicoureteral reflux (VUR) with single (*n* = 41) vs. recurrent episodes of UTIs (*n* = 42). The study also demonstrated microbial and metabolic profiles associated with febrile UTI status for participants.

The remaining four studies analyzed stool microbiomes to investigate the role of gut microbiota in UTI development [3,25,26,27]. Using 16S rRNA gene sequencing of stool samples collected from 35 children (age < 3 years) who received antibiotic treatment for acute UTIs, Akagawa et al. [25] investigated the effect of long-term antibiotic prophylaxis on the gut microbiota. Paalanne et al. [26] and Hong et al. [3] analyzed the gut microbiota in UTI and control groups (with the latter specifically studying preterm infants) and identified specific gut-microbiome-associated signatures of increased UTI risk. Adding to these studies on gut microbiota, Urakami et al. also compared the gut microbiota profiles in infants with febrile UTI (fUTI) and healthy controls, aiming to identify microbiota patterns during infancy that could indicate increased fUTI risk [27].

Overall, while five studies [3,23,24,26,27] incorporated both patients and controls to study the microbial differences associated with infection, risk of infection, number of episodes of UTI, and febrile UTIs, the other three studies [21,22,25] focused solely on UTI patients, investigating the microbial characteristics and alterations either unique to these cases or linked to antibiotic treatment regimes.

**Table 1:** Characteristics of the included studies.

Author	Study Design	Sample Type	Population/Sample Size	Median Age (Months)	Comparison	Identified Microbes	Outcome Measures	
							Primary Outcome	Secondary Outcomes
Hong et al., 2024 [3]	Longitudinal case-control	Gut	53 children with UTIs, 98 controls	6.90	Sepsis or NEC controls	UTI: *Enterococcus*, *Staphylococcus*, *Klebsiella*, *Escherichia coli*, *Enterobacter*; Control: *Bacteroides*, *Lactobacillus*	Alterations in gut microbiota and fecal calprotectin levels and their association with the development of UTIs in preterm infants.	Differences in microbiota between UTI cases and controls.
Urakami et al., 2023 [27]	Prospective case-control	Gut	28 infants with UTIs, 51 controls	5	HC	UTI: *Escherichia*, *Shigella*; Control: *Bacteroides fragilis*	Abnormal development of gut microbiota during infancy increases the risk of developing fUTI.	Lower levels of beneficial bacteria, potential link to immune responses.
Choi et al., 2022 [21]	Prospective cohort	Urine	57 children with UTIs	<6	-	UTI: *Escherichia fergusonii*, *Klebsiella pneumoniae*, *Shigella flexneri*	Identification of uropathogens, could be applied to manage the febrile UTI.	Comparison with conventional culture, impact on management.
Akagawa et al., 2020 [25]	Cohort study	Gut	35 children with UTIs	5.2	-	UTI: Enterobacteriales (decreased in CAP group)	Lactobacillales and gut microbiota diversity decreased compared with the pretreatment level.	Relative abundance of bacterial orders.
Vitko et al., 2021 [24]	Cross-sectional	Urine	For 16S rRNA sequencing (VUR = 33 and HC = 16); for metabolomic analysis (VUR with UTI = 83, HC = 13)	4.7	HC	UTI: *Dorea*, *Escherichia*; Control: *Prevotella*, *Lactobacillus*	Differences in microbiota and metabolomic profiles between patients vs. controls. *Prevotella*, and *Lactobacillus* dominant uMB profiles were more prevalent in controls.	Variations in profiles and metabolites associated with recurrent UTIs and VUR pathology.
Marshall et al., 2021 [22]	Cross-sectional	Urine	118 children with UTIs	40.9	-	UTI: *Escherichia coli*, *Ezakiella*; Control: *Prevotella*, *Porphyromonas*	Concordance between conventional culture and 16S rRNA gene amplicon sequencing appears to be high.	Detection of non-uropathogens, species diversity.
Kinneman et al., 2020 [23]	Cross-sectional	Urine	85 children	10.3	HC	UTI: *Escherichia coli*, *Proteus mirabilis*; Control: Normal urogenital flora	Identification of signature urinary microbiota in patients with a standard culture-positive UTI.	Association of diversity with variables.
Paalanne et al., 2018 [26]	Prospective case-control	Gut	37 children with UTIs, 69 controls	6.2	HC	UTI: *Enterobacter*, *Escherichia coli*; Control: Peptostreptococcaceae	Differences in the intestinal microbiome at family and genus levels may imply that the gut environment is linked with the risk of UTI in children.	No significant difference in fecal lactoferrin and iron concentrations at the phylum level, but at the genus level, *Enterobacter* was more abundant in UTI patients, and Peptostreptococcaceae were more abundant in healthy subjects.

Abbreviations: UTI, urinary tract infections; VUR, vesicoureteral reflux; HC, healthy control; CAP, continuous antibiotic prophylaxis.

### 3.2. Variation of Microbial Alpha Diversity in Pediatric UTIs

The included studies reported a reduction in alpha diversity with UTI onset (UTI cases vs. controls) [3,23], as well as UTI severity [24]. The studies by Kinneman et al. [23] and Hong et al. [3] identified significantly reduced alpha diversity in the urinary and gut microbiomes of UTI patients, respectively. Also, Urakami et al. [27] found that both the Shannon and Chao indices were significantly lower in the gut microbiota of the fUTI group than in healthy controls, suggesting that less diverse gut microbiota may increase the risk of infection. Additionally, Vitko et al. [24], analyzing the longitudinal changes in the urinary microbiome diversity, demonstrated a significant positive association between reduced diversity and increased UTI risk over time. Akagawa et al. [25] also evaluated the impact of antibiotic prophylaxis on gut microbiota, finding specific shifts in microbial groups without significant changes in overall diversity. Notably, the study by Paalanne et al. [26] reported mixed results, with some variations in diversity observed between UTI patients and controls.

### 3.3. Microbial Taxa Association with UTIs

**Figure 2:**
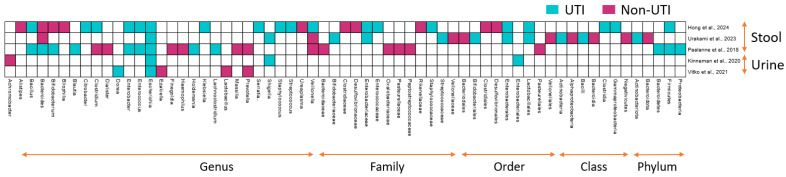
Comparative analysis of microbial profiles in UTI and non-UTI samples: heat map demonstrates distinct microbial signatures in UTI (blue colored) versus non-UTI (pink-red color) conditions across both stool and urine samples. UTI-associated samples, whether stool or urine, are characterized by an increased abundance of certain pathogenic microbes, while non-UTI samples exhibit a more balanced or diverse microbiota. This highlights the differences in microbial communities associated with UTI status and emphasizes the role of specific taxa in UTI pathogenesis [3,23,24,26,27].

Across the reviewed studies, *E. coli* consistently emerged as the predominant uropathogen in pediatric UTIs (Figure 2). Marshall et al. [22] reported that *E. coli* accounted for 95% of infections, and Choi et al. [21] found it in 90% of their cases. These findings highlight *E. coli* as a predominant pathogen in pediatric UTIs. In addition to *E. coli*, Choi et al. [21] identified other Enterobacteriaceae members like *Escherichia fergusonii*, *Klebsiella pneumoniae*, and *Shigella flexneri* in the urine of children with UTIs, expanding the range of pathogens potentially involved in infection. From the perspective of the gut microbiome, despite study-specific variations, all three studies, i.e., Hong et al. [3], Paalanne et al. [26], and Urakami et al. [27], demonstrated enrichment of *Escherichia*, *Shigella*, *Enterococcus*, *Enterobacter*, and *Bacillus* in the gut microbiota of the UTI patients, suggesting a link between gut dysbiosis and UTI risk (Figure 2).

In contrast to the disease-associated enrichment of the above taxa, the control-enriched taxa do not show consistencies across the studies, reflecting that UTIs are predominantly driven by the gain of specific “pathobionts”. The presence of certain genera, such as *Achromobacter*, *Alistipes*, *Ezakiella*, *Finegoldia*, *Haemophilus*, *Lactobacillus*, *Massilia*, *Prevotella*, *Bacteroides*, and *Ureaplasma*, predominantly in the gut of non-UTI individuals, was observed in the included studies (Figure 2). A greater microbiome diversity and the association of different core microbiome-associated genera in the gut of the non-UTI group emphasize the putative role of a diverse microbiome and diversity-associated core taxa in preventing infections.

### 3.4. Association of UTI with Host Variables

The microbiome composition profiles were also observed to be influenced by host-associated factors like age and sex. For example, Choi et al. [21] observed that children below 3 years of age had a higher prevalence of *E. coli* (85%) compared with older children (75%), indicating that age may influence UTI susceptibility. Hong et al. [3] found that preterm infants had elevated levels of *Enterococcus* and *Staphylococcus* in their gut microbiota, alongside a higher incidence of UTIs, which may be linked to an immature immune system and microbial dysbiosis. Urakami et al. [27] also noted a higher abundance of gut bacteria related to pathogenicity in infants with fUTI, highlighting early microbiota composition as a possible risk factor in pediatric UTI susceptibility. In addition, sex differences were also demonstrated by Kinneman et al. [23], who reported a higher prevalence of *Staphylococcus saprophyticus* in female children, a pathogen commonly associated with female UTIs. This finding aligns with the known anatomical and hormonal influences as females have a shorter urethra, which may facilitate easier bacterial entry and increase the risk of UTIs [28].

### 3.5. Functional Genomics and Metagenomics

Multi-omic analyses, particularly in the study by Vitko et al. [24], identified altered metabolic pathways linked to UTI pathogenesis. Changes in the glutamate degradation pathway were noted, with a shift in microbial communities leading to decreased levels of key metabolites like 2-oxoglutarate. This disruption in glutamate metabolism may influence microbial growth and activity, potentially exacerbating UTI conditions. Similarly, alterations in bile acid metabolism were observed, with increased production of secondary bile acids linked to changes in microbial composition. These metabolic changes can affect the urinary environment, making it more conducive to pathogen growth and contributing to the pathogenesis of UTIs.

### 3.6. Quality Assessment of Included Studies

**Figure 3:**
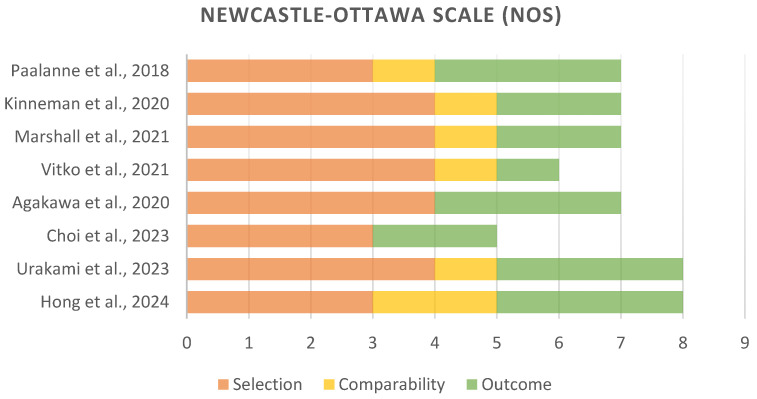
Methodological quality assessment using the Newcastle–Ottawa scale [3,21,22,23,24,25,26,27].

The included studies were evaluated for methodological quality using the NOS, with the findings revealing varying levels of methodological quality across study designs (Figure 3). The cohort study by Vitko et al. [24] was assigned a score of 6, reflecting strengths in the representativeness of the exposed cohorts, ascertainment of exposure, and comparability based on design or analysis. However, this study was limited by an inadequate follow-up duration and follow-up completeness, which may impact the robustness of the outcome assessments. Akagawa et al. [25] found that antibiotic prophylaxis led to specific shifts in microbial groups without significantly affecting overall diversity. However, it also had some limitations in comparability based on design or analysis. Cross-sectional studies by Kinneman et al. [23] and Marshall et al. [22] were assigned a score of 7 each, indicating strong performance in study design, sample representativeness, and statistical testing. These studies effectively demonstrated the outcome of interest and had comparability between groups. The study by Choi et al. [21] scored 5, with identified weaknesses in the representativeness of the sample and non-included subjects, which could influence the reliability of their findings. The case-control studies by Hong et al. [3] and Urakami et al. [27] were assigned a score of 8, reflecting strong methodological quality in case definition, representativeness, control selection, and exposure ascertainment.

## 4. Discussion

This systematic review investigates the association of gut and urinary microbiome profiles with the pathogenesis of pediatric UTIs, summarizing the consistent patterns of microbiome associations with UTI risk in children and their implications for UTI susceptibility and management. The most consistent finding was a loss of microbiome alpha-diversity and an enrichment of specific pathobiont microbes. This finding is potentially a reflection of the resilience-associated normal microbiome and an increase in dominance by specific pathogens, as has been observed for other diseases like inflammatory bowel disease [29,30].

In the included studies, *E. coli*, *K. pneumoniae*, and *S. flexneri* were consistently linked with UTIs [2,27,31]. Alternate pathogens like *Proteus mirabilis*, known for its urease activity that increases urine pH, and *Klebsiella pneumoniae*, capable of biofilm formation, present distinct challenges in UTI management. These microorganisms not only resist host immune responses but also modify the local microbial environment, promoting infection-prone conditions [32,33]. Similarly, pathogenic *Enterococcus* species can alter the local environment to enhance their survival and colonization, promoting an infection-prone setting [34].

Another crucial insight in this review is the list of microbial genera such as *Achromobacter*, *Lactobacillus*, *Ezakiella*, *Prevotella*, *Bacteroides*, and *Ureaplasma* that are enriched in the urine microbiome of the non-UTI children. This specific group of genera with potential protective roles against UTIs may be further explored as therapeutic agents against pediatric UTIs. The increased abundance of Lactobacilli in non-UTI patients, as documented by Paalanne et al. [26], is in line with previously reported associations of this lineage with improved host health in the gut and vaginal microbiomes. *Lactobacillus* species exert a protective effect against uropathogens such as *E. coli* [7]. This protective role is mediated through several mechanisms: the production of lactic acid lowers the pH of the urinary tract, creating an inhospitable environment for many pathogens, and hydrogen peroxide produced by *Lactobacillus* has antimicrobial properties that further inhibit pathogenic growth [35]. A high *Lactobacillus* population thus plays a vital role in maintaining a microbiome that resists pathogenic invasion. From the perspective of the gut microbiome as well, core members like *Prevotella* can potentially compete with *E. coli* for resources and thereby can protect against the colonization of these pathobionts in the gut [36,37].

Interestingly, a consistent pattern of gut microbiome alterations positively associated with UTI is the enrichment of pathobiont lineages of *Escherichia*, *Enterococcus*, *Enterobacter*, *Shigella*, and *Bacillus*, some of which have been previously established to cause urinary tract infections. The gut-associated enrichment of these pathogenic lineages with UTI onset or severity could putatively indicate microbial translocation from the gut to the urinary tract, particularly under conditions of gut dysbiosis [38]. However, these aspects need to be functionally and mechanistically validated using strain-level investigations on much larger cohorts. Furthermore, many of these UTI-enriched lineages in the gut microbiome, like *Enterobacter*, *Enterococcus*, and *Bacillus*, have also been reported to be consistently enriched in other infections like COVID-19 [39], indicating that these bacteria can be the putative markers of an infection-associated gut microbiome state and as “agitators” can detrimentally impact the host susceptibility to different infections.

Another crucial aspect is the longitudinal microbiome shift associated with UTI development. The findings of Vitko et al. [24] emphasize the dynamic nature of the urinary microbiome and its possible role in both the onset and resolution of UTIs. Although Vitko et al. [24] did not report specific Shannon index values, they demonstrated that reductions in microbial diversity consistently preceded UTI onset. Also, the longitudinal design of this study allowed for the observation of microbial shifts over time, linking these changes to clinical outcomes. This temporal perspective offers valuable insights into how variations in microbial diversity can correlate with UTI occurrences and can be translated into clinical practice for enhancing UTI risk assessment and patient monitoring. Routine assessments of microbial diversity could aid in stratifying patients by risk level, enabling more targeted strategies to prevent recurrent infections. Such measures can also be used to measure the efficacy of therapeutic interventions.

From the perspective of therapeutic interventions, the potential for probiotics and prebiotics is also noteworthy. Strains of probiotic lineages like *Lactobacillus*, which have demonstrated protective effects against uropathogens, could be explored for their preventive benefits [40,41]. For example, incorporating these probiotics into daily regimens might help restore beneficial microbial communities in the urinary and gut microbiomes, potentially reducing UTI incidence and severity. Prebiotics like inulin and fructooligosaccharides, which selectively promote the growth of beneficial bacteria, could also be included in dietary recommendations to support a balanced microbiome [42]. Similarly, prebiotics that selectively promote beneficial bacteria could support a balanced microbiome, contributing to UTI prevention. Additionally, developing guidelines for the appropriate use and dosage of these interventions based on patient-specific factors, such as age and underlying health conditions, could enhance their effectiveness.

The utilization of either the urinary or the gut microbiome or both for the management of pediatric UTIs is currently, however, a distant goal. Several limitations must be acknowledged in this regard and must be addressed as part of future studies. First, the variability in study designs, from cross-sectional to case-control and cohort studies, introduces heterogeneity that complicates evidence synthesis. Second, to date, the number of studies investigating either the urinary or the gut microbiome in pediatric UTI is just a few. This lack of sufficient published literature prevented us from performing a quantitative subgroup analysis. Third, we do not have a clear picture of what defines a “healthy” urobiome across diverse pediatric populations nor how it matures over time. Also, there is a lack of data on longitudinal profiling of urinary or gut microbial signatures in children with UTIs. There is a need for larger, multicentric longitudinal studies to validate the association between microbial diversity and UTI risk across diverse populations. These studies should employ standardized methodologies to enhance the comparability and generalizability of results. Moreover, there are currently no studies that have performed simultaneous longitudinal investigations of the urinary and gut microbiome from the perspective of UTI risk/severity. There is thus a need for studies performing an integrated investigation of the gut and urine microbiome along with other multi-omic profiles in relation to UTI risk and severity. Finally, it is important to understand the remote associations between the gut and the urobiome and how these putative interactions can either protect or predispose the host toward UTIs and utilization of gut and urine microbiome-associated markers for the management and surveillance of pediatric UTIs. Strain-level metagenomics and systems biology approaches could offer more comprehensive views of microbial ecosystems and the putative mechanisms of cross-body site interactions, paving the way for the development of personalized and targeted therapeutic interventions.

In this regard, further research is required to explore the mechanisms through which probiotics and prebiotics influence microbial balance and UTI susceptibility. This includes investigating the optimal strains, dosages, and duration of treatment to maximize therapeutic benefits and generate population-specific recommendations. Clinical trials are needed to refine these recommendations and establish best practices for integrating microbiome-focused therapies into routine care. Targeted metabolic interventions that can correct dysbiosis related to altered metabolism also present new treatment possibilities. In these lines, studies should examine the role of microbial metabolites and their impact on UTI pathogenesis to develop targeted metabolic interventions. Last, there is a need for research focusing on personalized approaches to UTI prevention and treatment, considering individual variations in microbiome composition and responses to interventions.

## Data Availability

Not applicable.

## Appendix A

**Table A1:** Details of the search strategy.

Step	Search String	No. of Studies
Database search: PubMed	((((((microbiome) OR (microbiota)) OR (microbe)) OR (microorganism)) OR (dysbiosis)) AND ((((gut) OR (fecal)) OR (urine)) OR (urinary))) AND ((“urinary tract infections”) OR (UTI))	116
Database search: Embase	(‘microbiome’/exp OR microbiome OR ‘microbiota’/exp OR microbiota OR ‘microbe’/exp OR microbe OR ‘microorganism’/exp OR microorganism OR ‘dysbiosis’/exp OR dysbiosis) AND (‘gut’/exp OR gut OR fecal OR ‘urine’/exp OR urine OR urinary) AND (‘urinary tract infections’/exp OR ‘urinary tract infections’ OR uti) AND [english]/lim AND ([newborn]/lim OR [infant]/lim OR [child]/lim OR [preschool]/lim OR [school]/lim OR [adolescent]/lim) AND [humans]/lim AND [2003–2023]/py	407
Database search: Web of Science	((((((microbiome) OR (microbiota)) OR (microbe)) OR (microorganism)) OR (dysbiosis)) AND ((((gut) OR (fecal)) OR (urine)) OR (urinary))) AND ((“urinary tract infections”) OR (UTI)) AND (Children)	201
Database search: Scopus	(microbiome OR microbiota OR microbe OR microorganism OR dysbiosis) AND (gut OR fecal OR urine OR urinary) AND (“urinary tract infections” OR uti) AND (children) AND PUBYEAR > 2002 AND PUBYEAR < 2024 AND (LIMIT-TO (EXACTKEYWORD, “Humans”)) AND (LIMIT-TO (LANGUAGE, “English”))	4791
Database search: total studies		5515
Database search: duplications		406
Database search: final articles screened		5109
Database search: full text reviewed		12
Database search: full text included		7
Additional sources: studies included		1
Final included in the systematic review		8
